# Evaluation of digital construction, production and intraoral position accuracy of novel 3D CAD/CAM titanium retainers

**DOI:** 10.1007/s00056-022-00393-8

**Published:** 2022-03-31

**Authors:** S. Koller, R. B. Craveiro, C. Niederau, T. L. Pollak, I. Knaup, M. Wolf

**Affiliations:** Department of Orthodontics, Dental Clinic, University Hospital RWTH Aachen, Pauwelsstr. 30, 52074 Aachen, Germany

**Keywords:** Computer-aided design/computer-aided manufacturing retainer, Fixed lingual retainers, Orthodontic treatment, Three dimensions, Long-term retention, Computer-aided design/computer-aided manufacturing Retainer, Festsitzende linguale Retainer, Kieferorthopädische Behandlung, Drei Dimensionen, Langfristige Retention

## Abstract

**Objectives:**

New opportunities have arisen to manufacture three-dimensional computer-aided design/computer-aided manufacturing (3D CAD/CAM) retainers from titanium blocks by digital cutting technology. These novel technologies need to fulfill requirements regarding digital planning and position accuracy. The aim of the present study was to investigate the digital construction, the CAD/CAM production and the intraoral positioning accuracy of custom-manufactured novel 3D CAD/CAM titanium retainers.

**Materials and methods:**

A total of 37 prime4me® RETAIN3R (Dentaurum, Ispringen, Germany) retainers were inserted to stabilize the upper anterior front teeth. Following insertion, an intraoral scan was used to record the position. The intraoral position was compared to the virtual setup using 3D superimposition software. Measurement points were evaluated in all three dimensions (horizontal, sagittal and vertical planes). Data were analyzed using Kruskal–Wallis test followed by Dunn’s multiple comparison test.

**Results:**

A total of 185 measurements were performed. The horizontal plane and the sagittal plane demonstrated a high level of positioning accuracy between the planned and the intraoral position. Statistically significant deviations between the preceding virtual setup and the intraoral situation were observed in the vertical dimension. Within the retainer, the intraoral positioning accuracy decreased for the measurement points in the direction of the distal retainer segment.

**Conclusion:**

Based on the results, the present study shows a high level of congruence between the 3D virtually planning and the final intraoral position of the fabricated novel 3D CAD/CAM titanium retainers.

## Abstract (de)

**Zielsetzungen:**

Gegenwärtig eröffnet die digitale Schneidetechnik neue Möglichkeiten zur Herstellung von 3‑D-CAD/CAM(dreidimensionaler „computer-aided design/computer-aided manufacturing“)-Retainern aus Titanblöcken. Diese neuen Technologien müssen die Anforderungen an die digitale Planung und die Positionsgenauigkeit erfüllen. In der vorliegenden Studie wurden die digitale Konstruktion, die CAD/CAM-Fertigung und die intraorale Positionierungsgenauigkeit von individuell gefertigten neuartigen 3‑D CAD/CAM-Titan-Retainern untersucht.

**Material und Methoden:**

Insgesamt 37 prime4me® RETAIN3R (Dentaurum, Ispringen, Deutschland) Retainer wurden zur Stabilisierung der oberen Frontzähne eingesetzt. Nach dem Einsetzen wurde die Position mit einem intraoralen Scan erfasst. Mithilfe einer 3‑D-Superimpositionssoftware wurde die intraorale Position mit der virtuellen Darstellung verglichen. Die Messpunkte wurden in allen 3 Dimensionen (horizontale, sagittale und vertikale Ebene) ausgewertet. Die Daten wurden mit dem Kruskal-Wallis-Test und anschließend mit dem Dunn-Mehrfachvergleichstest analysiert.

**Ergebnisse:**

Insgesamt wurden 185 Messungen durchgeführt. In der horizontalen Ebene und in der Sagittalebene zeigte sich eine hohe Positionierungsgenauigkeit zwischen der geplanten und der intraoralen Position. In der vertikalen Dimension wurden statistisch signifikante Abweichungen zwischen der virtuellen Darstellung und der intraoralen Stellung festgestellt. Im Retainer verringerte sich die intraorale Positionierungsgenauigkeit für die Messpunkte in Richtung des distalen Retainersegments.

**Schlussfolgerung:**

Die Ergebnisse der vorliegenden Studie zeigen eine hohe Kongruenz zwischen der virtuellen 3‑D-Planung und der endgültigen intraoralen Position der neuartigen 3‑D CAD/CAM-Titan-Retainer.

## Introduction

Long-term retention following orthodontic treatment has been a central issue in orthodontics for decades. There are ongoing discussions on multiple theories how permanent stabilization of orthodontic treatment can be addressed. While in the early years of modern orthodontics, Edward H. Angle advocated a secured neutral occlusion relationship as an effective means to stabilize tooth positions [1, 2], Charles Tweed postulated the avoidance of overexpansion of the dental arch by his “extraction for prevention” concept [3]. Some orthodontists claim that depending on the facial pattern using cephalometry, an individual position of the mandibular incisors needs to be defined to achieve long-term stability [4]. Others suggest an overcorrection of the present malocclusion or of tooth rotations [5]. In contrast, Reitan et al. advocated a concept of severing periodontal fibers to effectively prevent posttreatment displacement of teeth [1, 2, 6, 7]. However, recent studies have shown a tendency of lower anterior teeth to relapse into their previous malocclusion, if no appropriate retention devices for long-term stabilization have been incorporated [2, 8, 9].

Fixed retainers and removable appliances are the most common treatment options to prevent orthodontic relapse and tertiary crowding [2, 10].

Fixed lingual retainers have become an important factor in orthodontic retention due to the independence on the patient’s compliance, their simple fabrication combined with the fulfillment of high esthetic claims and especially the higher efficiency in maintaining incisor alignment compared to removable retainers, [6, 11–14]. Now, permanent lingual fixed stabilization in the esthetic zone has gained even more importance in modern orthodontics [2]. Particularly two different designs of fixed retainers have become established in orthodontics in recent years. First, round stainless-steel wire retainers bonded to the canines only [2, 15, 16] and second, multistranded wire retainers, as introduced by Zachrisson in 1977 [17, 18]. The multistranded steel wire retainers are usually bonded to all six front teeth of the upper or lower jaw and currently are considered the gold standard in orthodontic retention [10, 19–21]. Despite the benefit regarding tooth stabilization, the multistranded steel wire retainer can be associated with undesired side effects, such as self-inflicted tooth movements (x-effect, twist effect), retainer fractures, bonding-site defects, limited access to hygiene instruments as well as limited patient comfort [6, 8, 10, 15, 16, 20, 22]. Whereas round stainless-steel wire retainers bonded only to the canines are a reliable method for lower anterior teeth retention, they appear to be insufficient for the upper jaw because of their higher diameter and passive stabilization character. However, as most patients expect a lifelong stable tooth position after active orthodontic treatment, reliable permanent retention procedures have also become important in the upper jaw [22].

In that context, novel computer-aided designed and computer-aided manufactured (CAD/CAM) retainers, appear to be an innovative alternative to multistranded lingual retainers in the upper jaw. All of these take advantage of a precise and customized computer-aided design process. However, the possible production methods are highly diverse and range from robotic bending techniques to CNC (computerized numerical control) milling, laser or waterjet cutting, and metal laser sintering. Some of these methods are limited to a horizontal design. Especially CAD/CAM retainers with a three-dimensional individual design are able to consider potentially limited space in the maxilla and combine all benefits of retention appliances. For instance, a high level of patient comfort, thin bonding-sites, reduced limitation on oral hygiene, long-term stability, less side effects, reduced bonding-site defects and fracture rates, as well as reliable stabilization of the esthetic zone in the anterior maxilla [23]. According to the manufacturer, a virtual draft of the grade 5titanium CAD/CAM retainer considering all three dimensions is designed at first, before being cut from a grade 5 titanium blank. For this reason, for example the vertical dimension can be modified according to the clinical situation; thus, the retainer can be inserted even in anatomically adverse situations.

In the present study, we aim to evaluate the planning and positioning accuracy of a 3D CAD/CAM retainer by analyzing deviations between the virtual 3D design process and the clinical intraoral insertion.

## Methods

### Patients

In all, 37 fixed retainers, which had been inserted to stabilize preceding active orthodontic treatment outcomes, were analyzed in this study. All patients (20 females, 17 males) were treated by the same clinician. The study was performed in consent with the local ethics committee (EK 232-20). The study was conducted in full accordance of the ethical requirements of the World Medical Association Declaration of Helsinki (2008).

### Retainer fabrication

**Fig. 1:**
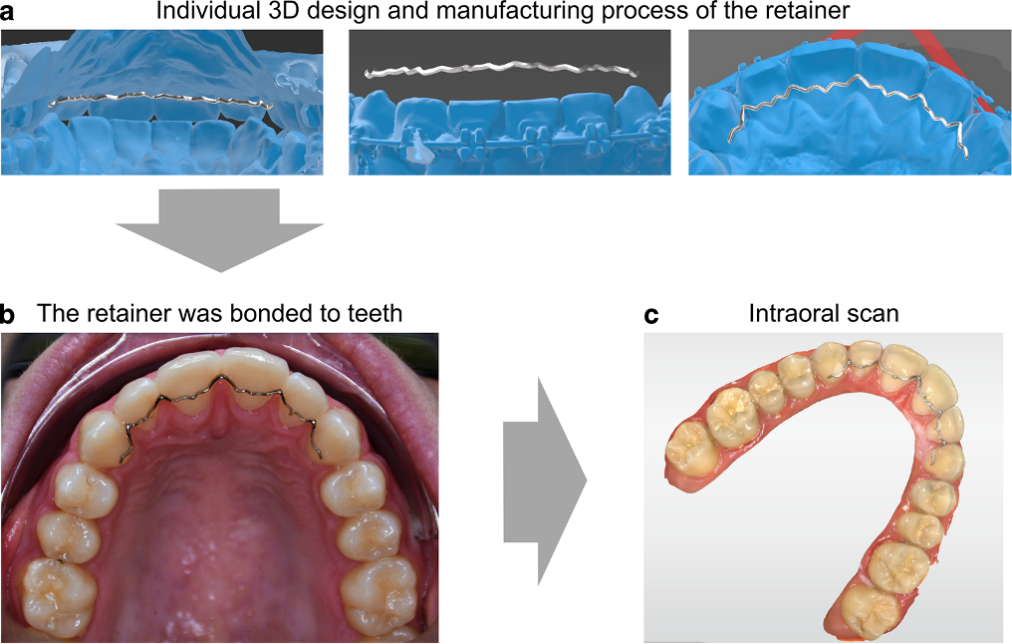
Retainer insertion. **a** Impressions of the individually and virtually designed retainer before the manufacturing process. **b** The retainer was adapted to the teeth using five threads and bonded using a light-curing low-viscosity composite resin. **c** A final intraoral scan following insertion was taken using the Primescan to record the definitive position Einsetzen des Retainers. **a** Abdrücke des individuell und virtuell gestalteten Retainers vor dem Herstellungsprozess. **b** Der Retainer wurde mit 5 Gewinden an die Zähne angepasst und mit einem lichthärtenden, niedrigviskosen Kompositharz verklebt. **c** Nach dem Einsetzen wurde ein abschließender intraoraler Scan mit dem Primescan angefertigt, um die endgültige Position festzuhalten

Intraoral scans of both the upper and lower jaw were taken including a bite registration, using the Primescan (version 5.1.1, manufactured 11/2019, DentsplySirona, Bensheim, Germany) after preceding orthodontic treatment and provision of written consent. Using STL datasets, the digital impressions were transferred to a commercial custom manufacturer of high-precision computer-aided design/computer-aided manufacturing (CAD/CAM) prime4me® RETAIN3R (Dentaurum, Ispringen, Germany). First, virtually designed drafts of the CAD/CAM retainers that took account the extension and position of the retainer were supplied by the manufacturer using screenshots (Fig. 1a). After confirmation of the virtual setup by the clinician, the prime4me® RETAIN3R was cut by a 5-axis milling machine from a titanium blank (grade 5) and polished by the custom manufacturer (KMZF Hostettler Dental AG, Huttwil, Switzerland). This production process allows an individual 3D design, including vertical adjustments. Subsequently, the retainer was sent to the orthodontic practice along with the STL dataset, which included the digital draft of the retainer.

### Retainer insertion

Each retainer included bonding sites on all maxillary incisors and canines, respectively, and were inserted by the same orthodontist. In order to achieve ideal bonding results on the maxillary anterior teeth, the incisors and canines initially were cleaned using a nonfluoride polishing paste (Omni clean and polish repair, OmniDent, Rodgau, Germany). After sandblasting the oral surfaces with aluminum oxide (50 µm, Airsonic® Alu-Oxyd, Hager Werken, Germany), the enamel surfaces were conditioned for 60 s using 37% phosphoric acid (smile Etch, smiledental, Ratingen, Germany), followed by rinsing with water and drying thoroughly. A thin bonding layer (Transbond XT, 3M, St. Paul, MN, USA) was then applied. Afterwards, the prime4me® RETAIN3R was adapted to the teeth using five threads. The fitting precision was checked with a dental probe and the retainer was bonded to all maxillary canines and incisors using a light-curing low-viscosity composite resin (Ortho Connect Flow, GC Orthodontics, Breckerfeld, Germany) (Fig. 1b). After checking the occlusal contacts and elimination of any premature contacts, a final intraoral scan was taken using the Primescan (Fig. 1c).

### Superimposition of the 3D models

**Fig. 2:**
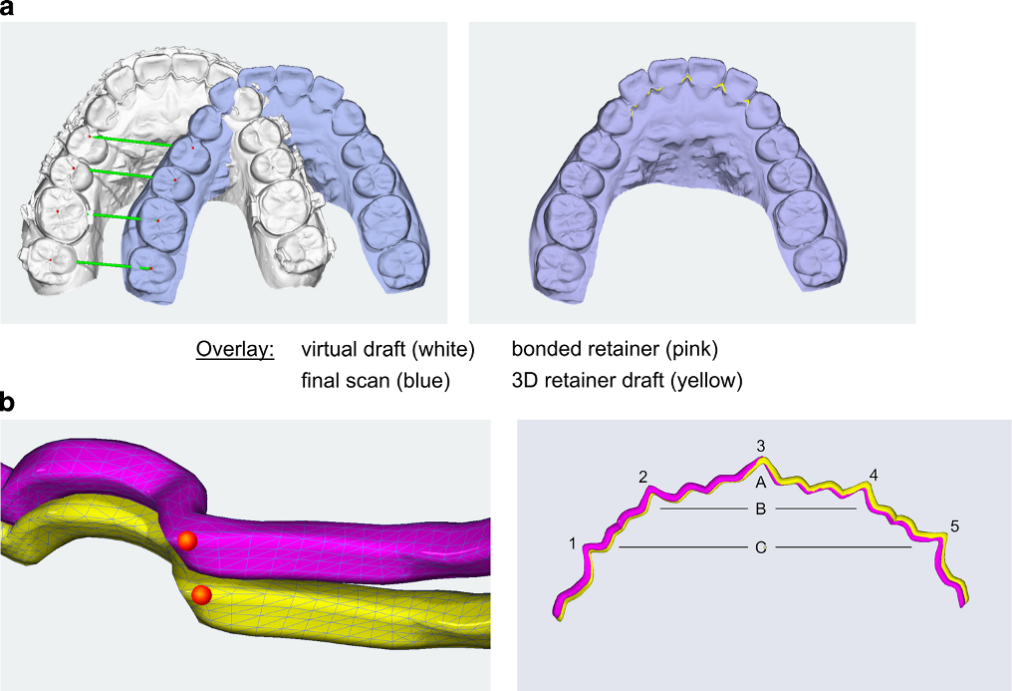
Superposition of the three-dimensional (3D) models and measurement. **a** Overlay of the final scan (*blue*), virtual draft (*white*), bonded retainer (*pink*) and 3D retainer draft (*yellow*) using multiple dental reference points in the posterior tooth region. **b** Definition of five interproximal measuring points on the wire Überlagerung der 3‑D(dreidimensionalen)-Modelle und Messung. **a** Überlagerung des endgültigen Scans (*blau*), des virtuellen Entwurfs (*weiß*), des geklebten Retainers (*rosa*) und des 3‑D-Retainerentwurfs (*gelb*) unter Verwendung mehrerer zahnmedizinischer Referenzpunkte im Seitenzahnbereich. **b** Definition von 5 interproximalen Messpunkten auf dem Draht

The intraoral retainer positions were compared by a 3D processing software (OnyxCeph, Image Instruments, Chemnitz, Germany) using an optimized superimposition protocol [2]. Joint crown reference points were defined in both final scans and 3D virtual drafts (Fig. 2a).

After superimposition of the virtual setup with the intraoral retainer position, deviations between the inserted retainers and the virtual setups were analyzed at all five interproximal reference points (Fig. 2b).

### Analysis of the retainer position

**Fig. 3:**
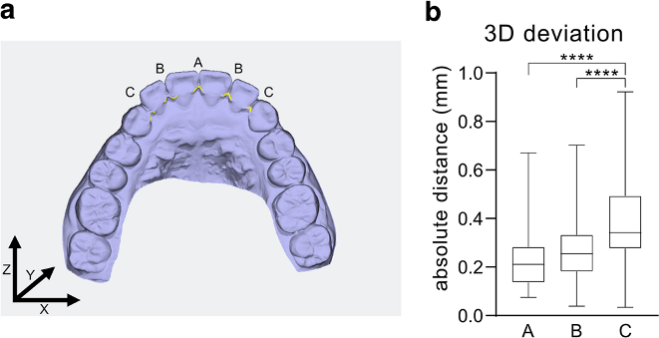
Deviations of the precision of positioning increase from the central measurement point to periphery of the retainer. **a** Definition of regions A, B and C and of x‑, y‑ and z‑axis. **b** Boxplot representations of the median (*black lines*), upper and lower quartiles (*boxes*) from 37 fixed retainers, which had been inserted to stabilize preceding active orthodontic treatment outcomes. Statistically significant differences are marked by *asterisks* (****p < 0.0001) according to the Kruskal–Wallis test Abweichungen der Positioniergenauigkeit nehmen vom zentralen Messpunkt zum Rand des Halters hin zu. **a** Definition der Regionen A, B und C sowie der x‑, y‑ und z‑Achse. **b** Boxplot-Darstellungen des Medians (*schwarze Linien*), des oberen und des unteren Quartils (*Kästen*) von 37 festsitzenden Retainern, die zur Stabilisierung vorangegangener aktiver kieferorthopädischer Behandlungsergebnisse eingesetzt worden waren. Statistisch signifikante Unterschiede sind durch *Asteriske* gekennzeichnet (****p < 0,0001) gemäß dem Kruskal-Wallis-Test

**Fig. 4:**
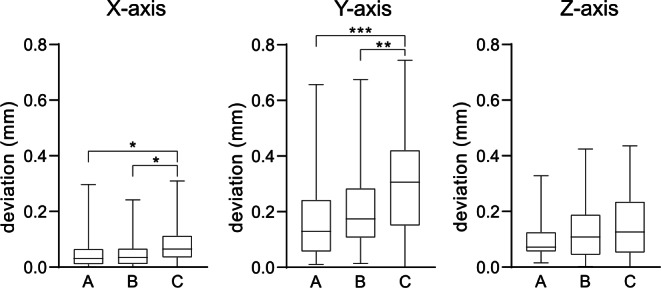
Deviation of retainer position x-, y‑ and z‑axis. Boxplot representations of the median (*black lines*), upper and lower quartiles (*boxes*) from 37 fixed retainers*. *Statistically significant differences are marked by *asterisks* (*p < 0.05; **p < 0.01; ***p < 0.001) according to the Kruskal–Wallis test Abweichung der Retainerposition auf der x‑, y‑ und z‑Achse. Boxplot-Darstellung des Medians (*schwarze Linien*), oberes und unteres Quartil (*Kästen*) von 37 fixierten Retainern. Statistisch signifikante Unterschiede sind durch *Asteriske* gekennzeichnet (*p < 0,05; **p < 0,01; ***p < 0,001) gemäß dem Kruskal-Wallis-Test

A total of 185 interproximal measurement points were analyzed for deviations between the final scans and the virtual setup regarding all three dimensions as described before [2]. In brief, each of the fixed retainers (*n* = 37) was analyzed in five interproximal measurement points (P1–P5). Point 1 (P1) describes the measurement point interproximal teeth 13/12, point 2 (P2) interproximal 12/11, point 3 (P3) interproximal 11/21, point 4 (P4) interproximal 21/22 and point 5 (P5) interproximal 22/23. The measure was divided in three regions: Point A describes the central measurement point, points B and C describe more peripheric points on the retainer according to P2/P4 and P1/P5, respectively (Fig. 3). First, the coordinates of the measuring points were exported and absolute deviations were calculated using the equation $\overline{AB}=\sqrt{{(x_{2}}-{x_{1}})^{2}+{(y_{2}}-{y_{1}})^{2}+{(z_{2}}-{z_{1}})^{2}}$ ($\overline{AB}$: absolute 3D distance; $x_{1}y_{1}z_{1}\colon$coordinates of the 3D Retainer dataset; $x_{2}y_{2}z_{2}\colon$ coordinates of the intraoral scan) (Fig. 3). Second, to determine the deviations within each axis, individual distances for the x‑, y‑, and z‑axis were calculated (Fig. 4). As shown in Fig. 3, the x‑axis represents lateral (horizontal), the y‑axis apicoincisal (vertical) and the z‑axis anterior–posterior (sagittal) deviations.

### Statistical analysis

The statistical analysis included the calculation of the mean distance and standard deviation between same measurement points (P1–P5) in space on the virtual setup and on the inserted retainer. In addition, mean deviations of the precision of positioning regarding all three dimensions for the same measurement points were calculated. Significance was determined using Kruskal–Wallis test followed by Dunn’s multiple comparison test due to a nonparametric distribution tested by Shapiro–Wilk test (Prism, version 8.1.0, GraphPad Software). Differences in retainer positions were regarded as statistically significant at *p* < 0.05. Absolute values were used for calculation of the deviations.

## Results

In all analyzed patients, we were able to digitize the upper dentition with the bonded 3D CAD/CAM retainer using direct intraoral scanning (Fig. 1) and to transfer the data to the analyzing and superimposition software. In all virtual models, the location of the upper retainers could be clearly identified, and no bonding irregularities were observed (Fig. 2).

The 3D CAD/CAM retainers showed an accurate 3D dimensional position where only a slight deviation could be observed for the measurement points in the distal retainer segments.

The close comparison of the virtual setup and the in situ retainer position indicated some absolute position differences between the digital and clinical situation at points A, B and C. Interestingly, an increase in position deviation could be observed towards the most distal points C. The differences between the virtual setup and the clinical situation at point A (median 0.211 [0.074/0.670]) and B (median 0.254 [0.038/0.702]) were statistically significant from that at points C (median 0.342 [0.033/0.922]) (Fig. 3a, b).

The superposition analyses of the virtual retainer setup and the intraoral inserted retainer position demonstrated some deviations in retainer position in all three dimensions. Furthermore, within the 3D analyses of retainer accuracy, the detected amounts of deviation increased the more distal they were located within the retainer. The smallest deviations were found in the horizontal plane (x-axis). Statistically significant horizontal deviations were measured between point A and point C as well as between point B and point C. Similar to the x‑axis, sagittal deviations were observed in anterior–posterior direction (z-axis), but did not reach the level of significance. The largest deviations between the virtual setup and the intraoral retainer could be measured in the vertical plane (y-axis) in the apicocoronal direction. Statistically significant deviations were measured between central point A and peripheric point C, as well as between lateral points B and C (Fig. 4a–c; Table 1).

**Table 1:** Absolute values and results of Kruskal–Wallis test followed by Dunn’s multiple comparison test due to a nonparametric distribution tested by Shapiro–Wilk test Absolute Werte und Ergebnisse des Kruskal-Wallis-Tests, gefolgt von einem Dunn-Mehrfachvergleichstest aufgrund einer nichtparametrischen Verteilung, geprüft mit dem Shapiro-Wilk-Test

Comparison		Median (min/max)	Mean	Mean Diff	Summary	Adjusted *P* value
*3D deviation *(Fig. 3)						
	A vs. B	A = 0.211 (0.074/0.670)	A = 0.2351	−0.0440	n. s.	0.1996
	A vs. C	B = 0.254 (0.038/0.702)	B = 0.2791	−0.1521	****	< 0.0001
	B vs. C	C = 0.342 (0.033/0.922)	C = 0.3872	−0.1081	****	< 0.0001
*Individual deviation in 3 axes *(Fig. 4)						
x‑Axis	A vs. B	A = 0.032 (0.002/0.297)	A = 0.0535	0.0001	n. s.	> 0.9999
	A vs. C	B = 0.034 (0.000/0.241)	B = 0.0533	−0.0316	**	0.0093
	B vs. C	C = 0.065 (0.000/0.310)	C = 0.0851	−0.0318	***	0.0008
y‑Axis	A vs. B	A = 0.129 (0.011/0.657)	A = 0.1746	−0.0364	n. s.	0.4896
	A vs. C	B = 0.174 (0.013/0.674)	B = 0.211	−0.1267	***	0.0002
	B vs. C	C = 0.306 (0.000/0.744)	C = 0.301	−0.0904	**	0.0035
z‑Axis	A vs. B	A = 0.071 (0.015/0.328)	A = 0.1026	−0.0226	n. s.	0.9925
	A vs. C	B = 0.108 (0.000/0.424)	B = 0.1251	−0.0444	n. s.	0.1737
	B vs. C	C = 0.126 (0.000/0.435)	C = 0.147	−0.0219	n. s.	0.7696

*n.s.* not significant

## Discussion

Nowadays, fixed lingual retainers represent a reliable way to permanently stabilize postorthodontic treatment results. Regarding the intraoral retention period of fixed retainers, a rethinking has taken place in modern orthodontics. Whereas previously an intermediate use of retainers was being advocated, recently an increasing number of clinicians consider a lifelong fixed retention as state-of-the-art for permanent tooth alignment [24–26]. For this purpose, fixed retainers need to fulfill certain requirements. These are the prevention of tooth movement, a high dimensional stability, no side effects, exact positioning of the wire and precision of fit, high patient comfort, accessibility for hygiene instruments and high biocompatibility. Custom made CAD/CAM 2D and 3D retainers seem to be a promising tool to optimize retention procedures in modern orthodontics.

In the past, we have published studies on the positioning accuracy of 2D retainers that did not allow for adjustability in the vertical dimension [2]. In this study, we used designs that allow customization in all planes, potentially allowing more precise adaptation to the dental arch and possibly clearer positioning. Based on our results, the present data confirm a precise clinical fit of the investigated 3D CAD/CAM process and a high clinical positioning accuracy. Due to the production method, we could provide evidence that the retainer adapted precisely to the oral surface morphology of the individual tooth and only minor deviations, without clinical relevance between the virtual planned and intraoral situation could be observed.

According to our measurements, only minimum deviations were found in the horizontal plane (x-axis), and at the central measurement point A between the central incisors. Interestingly, also in the 3D retainers the largest deviations between the virtual planning and the intraoral position were located in the vertical plane. This finding agrees with previous reports on 2D retainers, which also showed most discrepancies between the digital setup and intraoral positions in the vertical plane [2]. In addition, 3D retainers showed a trend to a decreased position accuracy towards the distal retainer segment. The weakness in positioning precision in the vertical compared to the sagittal and horizontal plane may be explained by the tooth anatomy. The precise design and manufacturing of the retainer makes it possible to achieve accurate positioning in the sagittal and horizontal planes. By extending the retainer into the approximal spaces, it may find its’ planned position precisely. In contrast, these characteristic structures are missing on the palatal tooth surfaces, which leads to a possible deviation in the vertical plane in particular. An improvement of the intraoral fitting precision could possibly be achieved by the insertion of the retainer with the help of a transfer jig [2, 15].

Taking the high precision of fit and material characteristics of the used 3D CAD/CAM retainer into account, it has to be evaluated in future investigations whether this precision will help to reduce undesired side effects associated with fixed retainers. In consideration of a study that investigated orthodontic retention procedures and that pointed out that unintentionally active fixed retainers leading to further orthodontic treatment were reported by over 30% of 300 questioned orthodontists, elimination of these effects is expected to be clinically highly relevant [27].

One of the advantages of individualized 3D retainers like in our study is that it allows a three-dimensional design. Especially in anatomically adverse situations, the vertical dimension of the retainer can be altered throughout the custom design and manufacturing process to prevent premature contacts. If the wire design and bonding zone morphology can be predicted beforehand, future studies need to evaluate a possible positive impact regarding bonding-site defects and retainer failure rate, as it has been frequently mentioned in literature [28, 29].

Clinically, high precision planning of intraoral bonding-site positions accounts for better oral hygiene and an increase in patient comfort due to thinner bonding-sites. Flatter and more delicate bonding sites along with a smooth surface texture of the CAD/CAM fabricated retainer had a positive impact on plaque and calculus accumulation due to an improvement of interproximal hygiene. In contrast, thicker bonding sites and a higher plaque and calculus accumulation were observed in multistranded wires [10, 30, 31].

Regarding the material characteristics, a high level of biocompatibility can be expected for the investigated 3D retainer because of the titanium grade 5 material [32]. This could be a good alternative for patients with nickel allergies. In addition, titanium retainers do not have ferromagnetic properties and therefore showed smaller scale MRI artifacts compared to round stainless-steel wire or multistranded wire retainers [24]. Similarly, the very rigid material of steel retainers limits the physiological mobility of the teeth, whereas this is made possible by the material properties of nickel titanium and titanium grade 5.

In summary, the present study revealed only minor deviations regarding the positioning precision between virtually planned 3D retainers and the clinical intraoral situation after retainer bonding. Clinically, the 3D retainers could be precisely adapted to the tooth surfaces and all retainers were inserted successfully in all cases. With a maximum of only 0.4 mm, the observed deviations were not clinically relevant. Further long-term studies are need to analyze the retainer in terms of failure rate, bonding-site defects and stabilization of treatment outcomes in order to define a concluding gold-standard for long-term retention.
